# Global insights into the genome dynamics of *Clostridioides difficile* associated with antimicrobial resistance, virulence, and genomic adaptations among clonal lineages

**DOI:** 10.3389/fcimb.2024.1493225

**Published:** 2025-01-15

**Authors:** Mohammad Sholeh, Masoumeh Beig, Ebrahim Kouhsari, Mahdi Rohani, Mohammad Katouli, Farzad Badmasti

**Affiliations:** ^1^ Department of Bacteriology, Pasteur Institute of Iran, Tehran, Iran; ^2^ Student Research Committee, Pasteur Institute of Iran, Tehran, Iran; ^3^ Laboratory Sciences Research Center, Golestan University of Medical Sciences, Gorgan, Iran; ^4^ Genecology Research Centre, Faculty of Science, Health, Education and Engineering, University of the Sunshine Coast, Maroochydore DC, QLD, Australia

**Keywords:** *Clostridioides difficile*, genomics, pan-genome analysis, sequence typing, plasmid, chromosome

## Abstract

**Background:**

*Clostridioides difficile* is a significant cause of healthcare-associated infections, with rising antimicrobial resistance complicating treatment. This study offers a genomic analysis of *C. difficile*, focusing on sequence types (STs), global distribution, antibiotic resistance genes, and virulence factors in its chromosomal and plasmid DNA.

**Methods:**

A total of 19,711 *C. difficile* genomes were retrieved from GenBank. Prokka was used for genome annotation, and multi-locus sequence typing (MLST) identified STs. Pan-genome analysis with Roary identified core and accessory genes. Antibiotic resistance genes, virulence factors, and toxins were detected using the CARD and VFDB databases, and the ABRicate software. Statistical analyses and visualizations were performed in R.

**Results:**

Among 366 identified STs, ST1 (1,326 isolates), ST2 (1,141), ST11 (893), and ST42 (763) were predominant. Trends of genome streamlining included reductions in chromosomal length, gene count, protein-coding genes, and pseudogenes. Common antibiotic resistance genes—*cdeA* (99.46%), *cplR* (99.63%), and *nimB* (99.67%)—were nearly ubiquitous. Rare resistance genes like *blaCTX-M-2*, *cfxA3*, and *blaZ* appeared in only 0.005% of genomes. Vancomycin susceptibility-reducing *vanG* cluster genes were detected at low frequencies. Virulence factors showed variability, with highly prevalent genes such as *zmp1* (99.62%), *groEL* (99.60%), and *rpoB/rpoB2* (99.60%). Moderately distributed genes included *cwp66* (54.61%) and *slpA* (79.02%). Toxin genes *tcdE* (91.26%), *tcdC* (89.67%), and *tcdB* (89.06%) were widespread, while binary toxin genes *cdtA* (26.19%) and *cdtB* (26.26%) were less common. Toxin gene prevalence, particularly *tcdA* and *tcdB*, showed a gradual decline over time, with sharper reductions for *cdtA* and *cdtB*. Gene presence patterns (GPP-1) for resistance, virulence, and toxin genes were primarily linked to ST2, ST42, and ST8.

**Conclusion:**

This study highlights *C. difficile*’s adaptability and genetic diversity. The decline in toxin genes reflects fewer toxigenic isolates, but the bacterium’s increasing preserved resistance factors and virulence genes enable its rapid evolution. ST2, ST42, and ST8 dominate globally, emphasizing the need for ongoing monitoring.

## 1 Introduction

*Clostridioides difficile* is a Gram-positive, spore-forming bacterium (Giles and Roberts, 2022). This obligate anaerobic bacterium is well-known for producing toxins that can cause antibiotic-associated diarrhea. *C. difficile* infection (CDI) is recognized globally as a serious health threat, particularly in vulnerable individuals (Etifa, 2021).

CDI is significant in older individuals, with high recurrence rates due to antibiotic disruption of the gastrointestinal microbiota (Asempa and Nicolau, 2017). Although antibiotics are essential for treating CDI, they can cause recurrence, prompting interest in alternative therapies, such as phage therapy and fecal microbiota transplantation (Phanchana et al., 2021).

*C. difficile* is considered a high-risk pathogen due to its extensive spectrum of antibiotic resistance (Kouhsari et al., 2019; Sholeh et al., 2020; Sholeh et al., 2021). This resistance makes it particularly challenging to treat and manage, significantly impacting healthcare settings (Sholeh et al., 2020; Doll et al., 2021; Buddle and Fagan, 2023). The Centers for Disease Control and Prevention (CDC) has classified *C. difficile* as a significant public health threat because of its increasing resistance to multiple antibiotics. This classification underscores the urgent need for new antimicrobial treatments to combat the evolving threat (Szymczak et al., 2020).

Genomic studies have revealed the complexity of *C. difficile*, highlighting significant genetic variations, antibiotic resistance genes, and virulence factors in both plasmids and chromosomes (Buddle and Fagan, 2023). These insights are critical for understanding how *C. difficile* causes disease and resistance to treatment (Dureja et al., 2022). Plasmids play a crucial role in this context by harboring genes that enhance virulence and resistance and contribute to pathogenic pathways (Boekhoud et al., 2020; Botelho and Schulenburg, 2021). Virulence factors, particularly toxins A and B, are key to the severity and recurrence of CDI, and genetic studies offer critical insights into the pathogen’s disease-causing ability and evasion of host defenses (Nibbering et al., 2021; Sholeh et al., 2021). The genetic elements of *C. difficile*, such as plasmids and chromosomes, are vital for the spread of antibiotic resistance and virulence genes. Understanding these mechanisms is essential for developing effective CDI therapies, particularly considering the challenges of multidrug resistance (Hornung et al., 2019). Bacterial resistance to multiple antibiotics complicates CDI treatment and highlights the need to elucidate the genetic basis of resistance (Fitzpatrick et al., 2022). Recent genomic studies have enhanced our understanding of *C. difficile*, revealing its genetic adaptations, antibiotic resistance mechanisms, and virulence factors, particularly by identifying diverse accessory genomes within different sequence types (STs) (Lewis et al., 2017). Hornung et al. conducted an *in-silico* survey that discovered numerous previously uncharacterized plasmids in *C. difficile*, suggesting that these extrachromosomal elements may contribute to the bacterium’s pathogenicity by carrying genes related to antibiotic resistance and virulence (Hornung et al., 2019). Current research on *C. difficile* has identified a significant gap, particularly in comprehensive analyses integrating findings on plasmids, pathogenicity, and resistance mechanisms across various STs and geographical regions. While some studies have focused on specific genes, there is a need for extensive genomic data on STs, global distribution, resistance genes, and virulence factors in chromosomal and plasmid DNA. This study aimed to fill these gaps by providing integrative genomic analyses of critical components of plasmids and chromosomes, including antimicrobial resistance genes and virulence factors. By mapping these genetic elements, this study sought to enhance our understanding of their roles in *C. difficile* pathogenicity and resistance, which is essential for developing effective therapeutic strategies.

## 2 Methods

### 2.1 Data acquisition

The complete genome sequence of *C. difficile* was retrieved from the GenBank database (GenBank) (https://www.ncbi.nlm.nih.gov/genbank/). Both plasmids and chromosomes were considered for separate analyses. **Supplementary Table 1** provides detailed information, including accession numbers, BioProject numbers, and metadata for the sequences.

### 2.2 Genome annotation

Both chromosomal and plasmid sequences were annotated using Prokka (version 1.14.6), a rapid annotation tool for prokaryotic genomes (Prokka GitHub) (Seemann, 2014). Prokka uses comprehensive pipelines and automated annotation to identify coding sequences, RNA genes, and other genomic features. Default settings were used, and annotations were verified against known databases to ensure accuracy and consistency.

### 2.3 Multi-locus sequence typing

MLST was conducted using the command-line tool MLST (version 2.19.0) (MLST GitHub) (Jolley and Maiden, 2010). This tool characterizes isolates based on internal fragment sequences of multiple housekeeping genes. The resulting allelic profiles were used to classify each *C. difficile* strain into specific STs to improve our understanding of genetic diversity and epidemiological patterns.

### 2.4 Pangenome analysis and clustering

Pangenome analysis and clustering were performed using Roary (version 3.13.0), a pipeline for analyzing prokaryotic pangenome (Roary GitHub) (Page et al., 2015). Roary determines the presence or absence of genes across different strains and helps define core and accessory genomes. Default parameters were applied, and the results were visualized to illustrate the genomic diversity of the *C. difficile* strains.

### 2.5 Identification of antibiotic resistance and virulence genes

The identification of antibiotic resistance genes and virulence factors, including specific *C. difficile-associated toxins* such as *tcdA*, *tcdB*, *tcdC*, *tcdE*, *tcdR*, *cdtA*, *cdtB*, and *cdtR*, was achieved through an integrated approach using both established and custom databases. The Comprehensive Antimicrobial Resistance Database (CARD) (https://card.mcmaster.ca/home) (version 3.2.5) (https://card.mcmaster.ca/home) (Jia et al., 2017) was used to detect resistance genes. In contrast, the Virulence Factor Database (VFDB, version 2023) (http://www.mgc.ac.cn/VFs/) (Chen et al., 2016) is a primary source of virulence genes. In addition, custom datasets were meticulously crafted to enhance the precision of detecting the critical *C. difficile* toxins. The ABRicate software tool was instrumental in this analysis, enabling thorough screening of genomic sequences and ensuring accurate identification of resistance genes and virulence factors, including specific toxins of interest (Chen et al., 2016).

### 2.6 Statistical analysis and visualization

The text with hyperlinks added to each package or software application is described as follows. To analyze the genetic data of *C. difficile* and examine the relationship between sequence length and year of isolation, we used the R package (version 4.3.1). Essential packages facilitate data handling, visualization, and sequence analysis. Pearson’s correlation coefficients were calculated using the Hmisc package (version 5.0-0) to assess trends between chromosomal sequence lengths and collection dates, and ggplot2 (version 3.4.0) was used to create scatter plots and trend lines. The repel package (version 0.9.1) improves plot readability by preventing label overlap. Data manipulation was streamlined using dpyr (version 1.1.2) and read (version 2.1.4), whereas the tidyverse (version 2.0.0) ensured a cohesive analysis workflow. Biostrings (version 2.70.1) provided robust tools for biological sequence operations, and Entrez (version 1.2.3) enabled efficient genomic data retrieval from NCBI. Complex datasets were visualized using Pheatmap (version 1.0.12) for heat maps and PHYLOViZ Version 2 to analyze and plot MLST data with associated resistance genes.

## 3 Result

### 3.1 Genomic characteristics, geographical distribution, and temporal trends of sequence types among *C. difficile*

We analyzed 19,711 whole-genome sequences of *C. difficile*, including 207 isolates at the complete genome level, 39 at the chromosome level, 1,918 at the scaffold level, and 17,547 at the contig level. On average, each isolate contained 3,915 annotated genes, including 3,778 protein-coding genes and 65 pseudogenes, with an average genome length of 4.2 Mbp. The N50 values were 272 Kbp for contigs and 657 Kbp for scaffolds. These isolates, spanning 56 countries from 2000 to 2024, were predominantly human-derived (16,303 isolates), with the remainder from non-human hosts (3,407 isolates). Detailed genomic data can be found in **Supplementary Data Sheet 1**.

**Figure 1:**
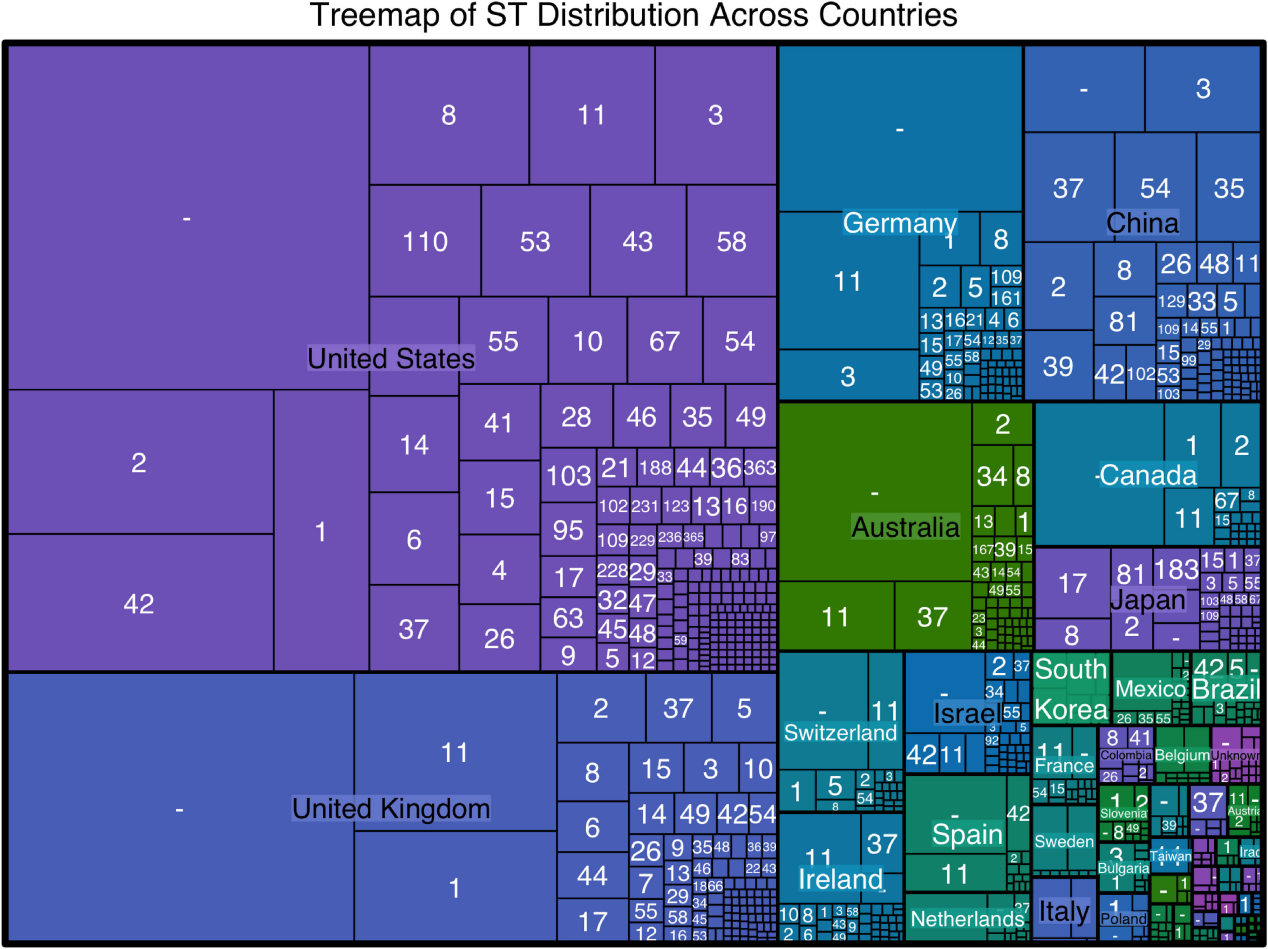
TreeMap visualizes the sequence type (ST) distribution across different countries. The size of each rectangle corresponds to the number of isolates of a particular ST in each country, highlighting the most prevalent STs within each region. The United States shows the highest diversity of STs, with ST34, ST1, and ST42 being the most common. Other countries with significant ST diversity include the United Kingdom, Germany, China, and Australia.

The analysis identified 366 distinct sequence types (STs) among 19,654 *C. difficile* isolates, reflecting significant genetic diversity. The most common STs were ST1 (1,326 isolates), ST2 (1,141 isolates), ST11 (893 isolates), and ST42 (763 isolates). North America, particularly the United States, exhibited the highest ST diversity with 195 unique STs, Europe with 125 STs, Asia with 105 STs, Oceania with 55 STs, and South America with 28 STs. Detailed geographical distributions are provided in **Supplementary Data Sheet 1**, with visual representations in **Figure 1**.

**Figure 2:**
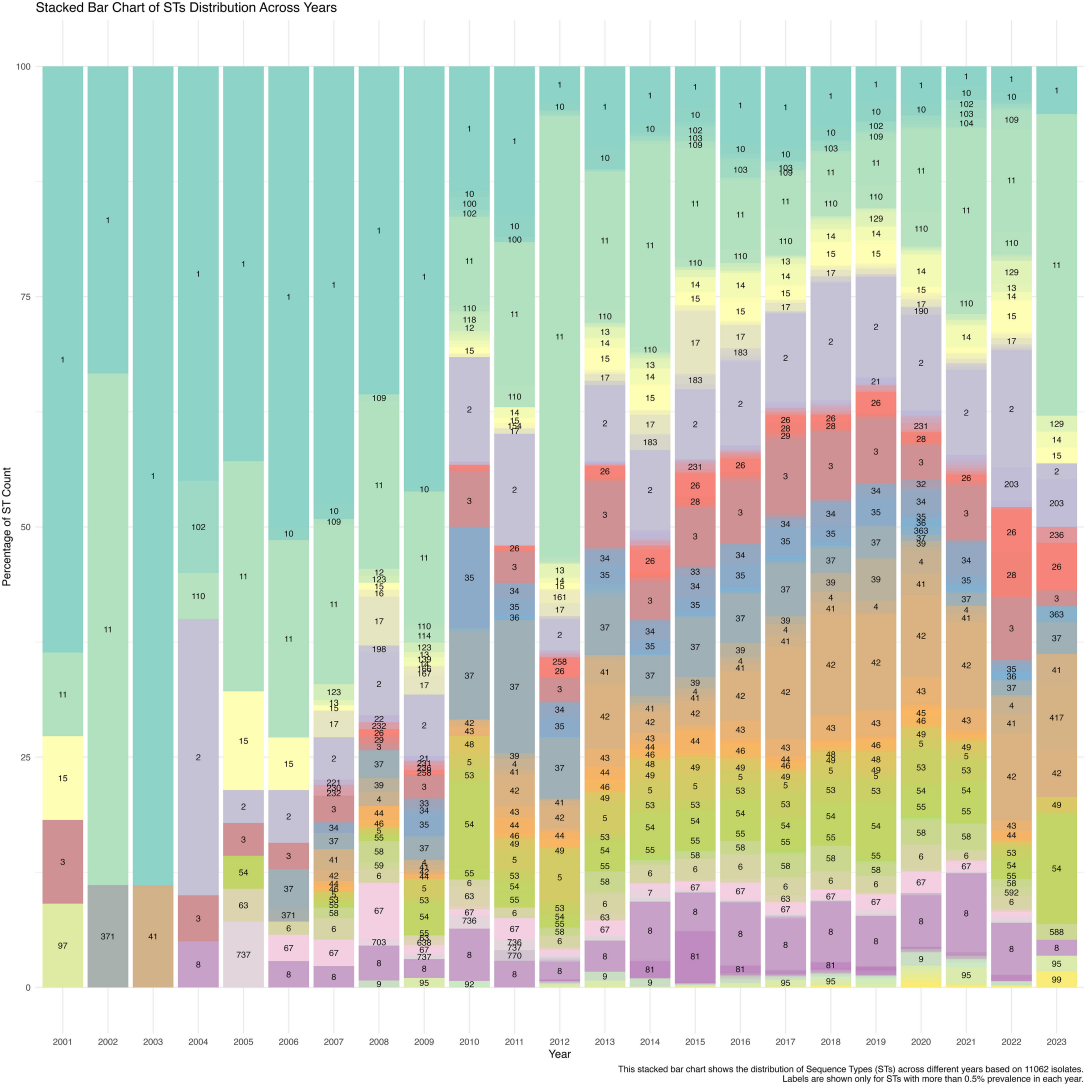
Minimum-spanning tree (MST) visualisation of STs and their geographic distribution, generated using PHYLOViZ analysis. Each node represents a different ST, with the node’s size proportional to the number of isolates. The connections between nodes indicate the genetic relatedness between STs, with closely related STs clustered together. The pie charts within each node display the ST distribution across various countries, providing insights into the global geographic spread and diversity of specific STs.

The temporal analysis over 23 years based on 11,062 isolates revealed a consistent prevalence of ST1 and ST11, with an increasing prominence of ST11 post-2010. The frequencies of other STs, such as ST37, ST42, and ST54, increased during the early 2010s, indicating shifts in the genetic landscape likely influenced by clinical practices, antibiotic use, and environmental factors. The data suggest a trend towards increasing ST diversity from 2010 onwards, highlighting a broad genetic pool within *C. difficile* populations (**Figure 2**).

**Figure 3:**
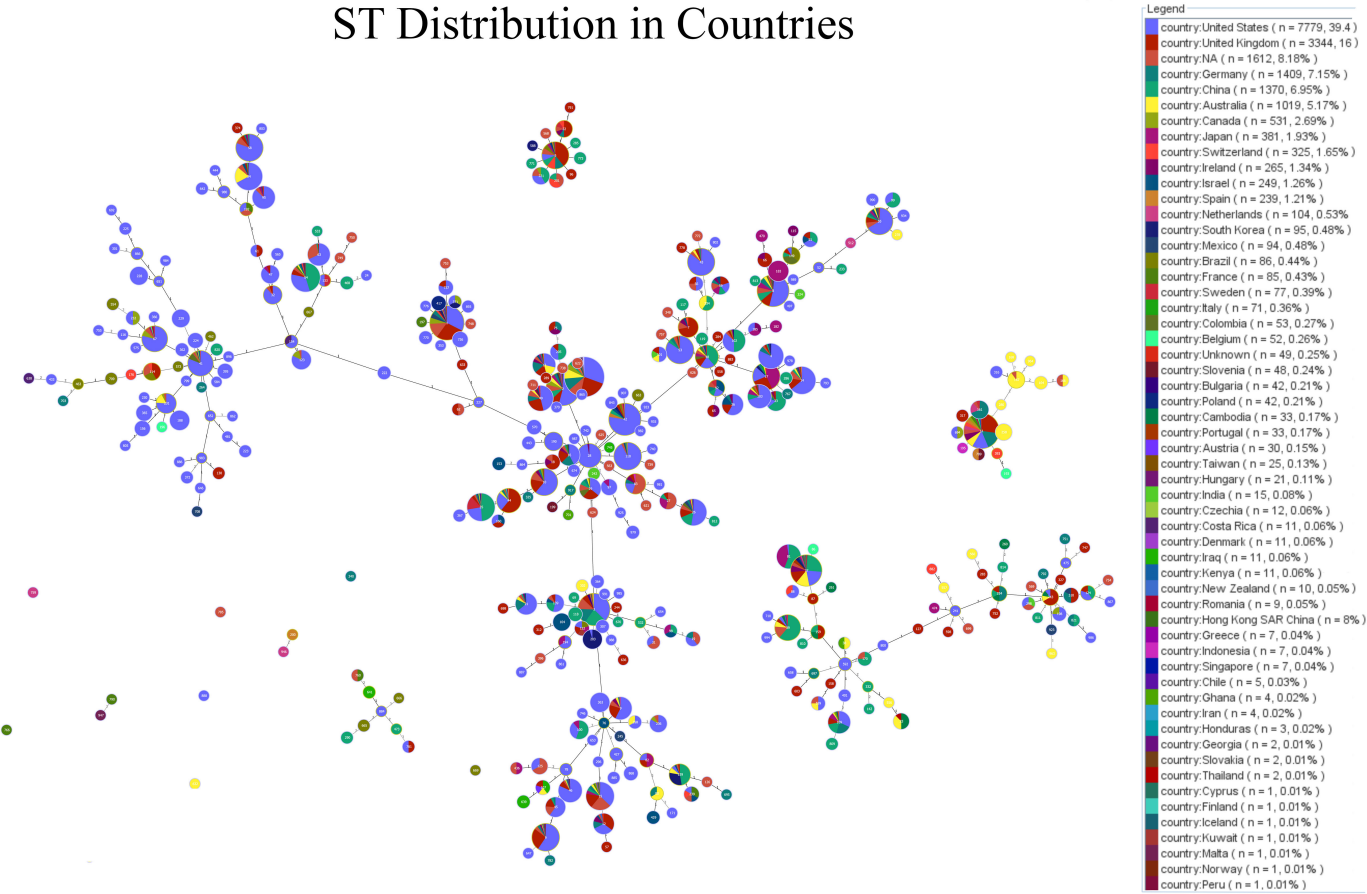
A stacked bar chart illustrates the distribution of Sequence Types (STs) across different years based on 11,062 C. difficile isolates. The labels show the STs with more than 0.5% prevalence each year, highlighting temporal trends in ST dominance and diversity from 2001 to 2023.

The minimum-spanning tree (MST) visualization of sequence types (STs) and their geographic distribution, generated using PHYLOViZ analysis, provides a detailed overview of the genetic relationships and global spread of *C. difficile* isolates. As shown in **Figure 3**, each node represents a distinct ST, with the size of the node proportional to the number of isolates. For instance, ST1 and ST2, the largest nodes, indicate a higher number of isolates, while smaller nodes represent less prevalent STs. The connections between nodes demonstrate the genetic relatedness of the STs, with closely related STs clustered together. Notably, ST1 is found to be widely distributed across Europe, North America, and Asia, while ST3 is predominantly present in Europe. The pie charts within each node provide additional insights into the geographic distribution, with countries like the United States, France, and the UK showing significant representation of specific STs. This MST analysis highlights the widespread presence of certain STs and their clustering based on genetic similarity, offering valuable information on the global geographic spread and diversity of *C. difficile* strains.

**Figure 4:**
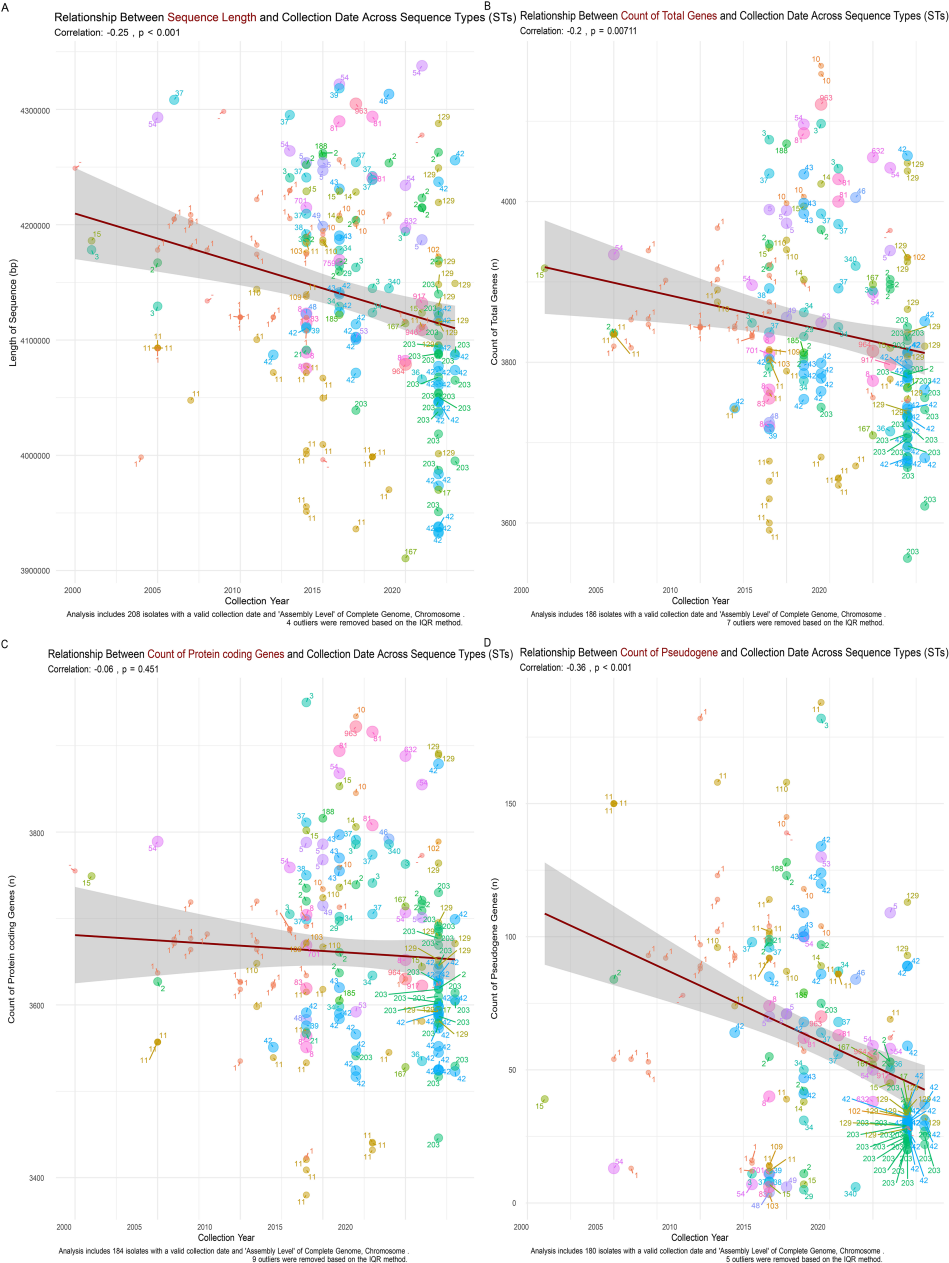
Relationship Between Sequence Metrics and Collection Date Across *C. difficile* Sequence Types (STs). **(A)** Chromosomal sequence length shows a significant negative correlation with collection date (r = -0.25, p < 0.001), indicating a trend of genome streamlining in more recent isolates. **(B)** Total gene count decreases over time (r = -0.2, p = 0.00711), reflecting a reduction in genomic content consistent with the shorter chromosomal sequences observed. **(C)** Protein-coding gene count shows a slight negative trend with collection year (r = -0.06, p = 0.451), suggesting the gradual loss of non-essential genes. **(D)** Pseudogene count exhibits a significant negative correlation with collection date (r = -0.36, p < 0.001), indicating the purging of non-functional genetic elements over time as part of the genome streamlining process.

Further analysis of sequence metrics across different STs showed a significant inverse relationship between chromosomal sequence length and collection year, with longer sequences, such as ST947 and ST54, possibly conferring adaptive advantages (**Figure 4A**). The total gene count (**Figure 4B**), protein-coding gene count (**Figure 4C**), and pseudogene count also exhibited negative correlations with collection year (**Figure 4D**), supporting the hypothesis of genome contraction and optimization in response to environmental pressures. **Supplementary Figure 1** provides additional details on sequence type length trends.

### 3.2 Pangenome analysis of *C. difficile*

Pan-genome analysis of 239 C*. difficile* isolates, specifically those with assembly levels classified as complete genomes or chromosomes, revealed substantial genetic diversity and adaptability within the species. The pan-genome comprised 16,329 genes, which were categorized into four distinct groups: the core genome, containing 1,670 genes found in 99-100% of strains; the software genome, comprising 517 genes present in 95-99% of strains; the shell genome, comprising 2,278 genes occurring in 15-95% of strains; and the cloud genome, containing 11,864 genes found in 15% of strains. The core genome is responsible for essential cellular functions, which are conserved across all strains. In contrast, shell and cloud genomes highlight the remarkable capacity of these species to adapt to diverse environments and ecological niches. **Supplementary Figure 2** illustrates this distribution, emphasizing the extensive variability in non-core genes, likely contributing to the bacterium’s evolutionary flexibility and environmental success.

### 3.3 Antibiotic resistance in *C. difficile*

**Figure 5:**
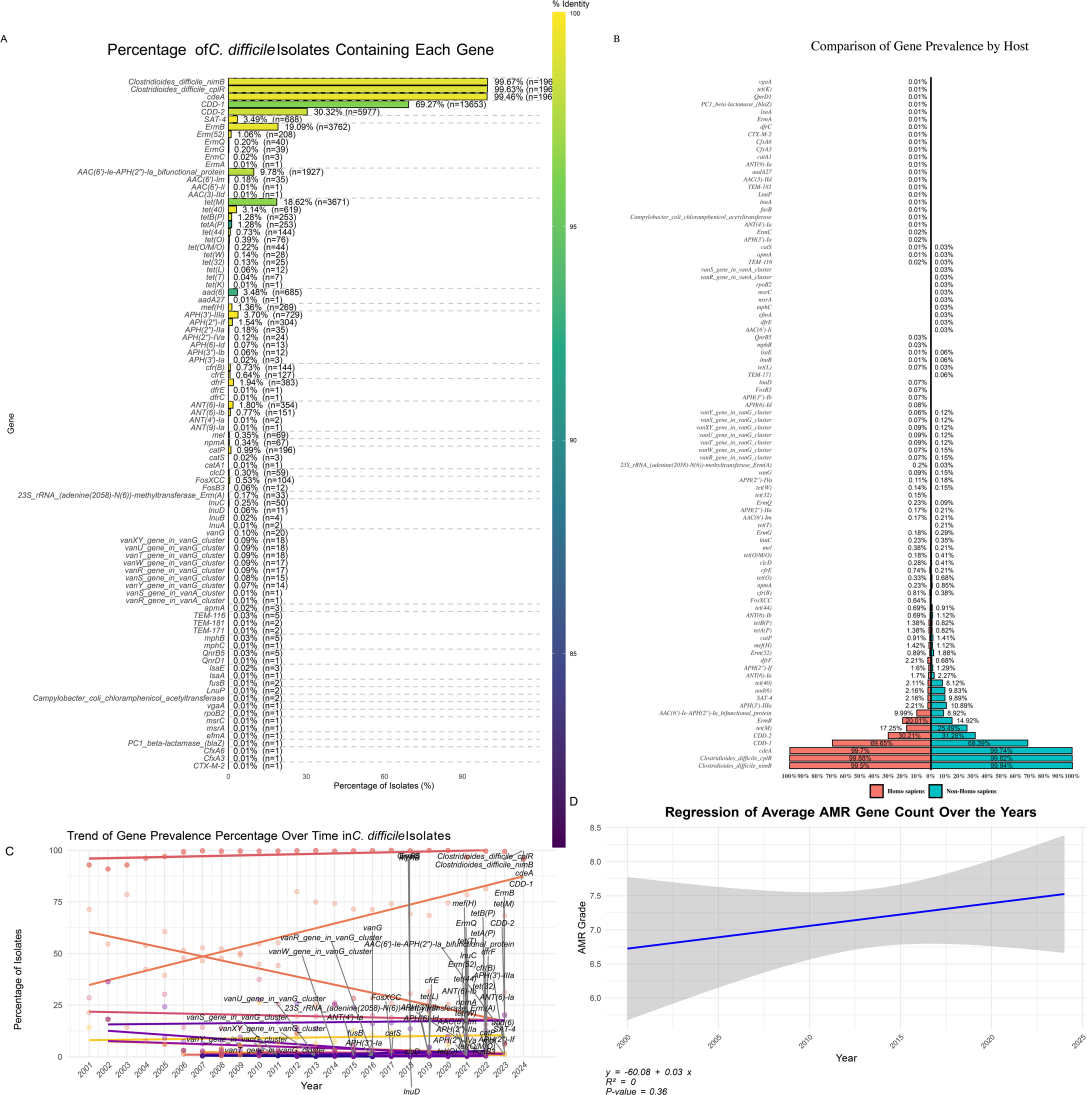
Distribution of Antibiotic Resistance Genes in *C. difficile* Isolates. **(A)** Percentage of *C. difficile* Isolates Containing Each Gene: Bar plot illustrating the prevalence of specific antibiotic resistance genes among *C. difficile* isolates. The colour gradient represents the percentage of isolates containing each gene, highlighting the most and least common resistance genes. **(B)** Distribution of Gene Prevalence by Host: A bar plot showing the percentage of gene prevalence grouped by different hosts compares the occurrence of resistance genes in human versus non-human hosts. **(C)** Trend of Gene Prevalence Percentage Over Time: Line plot demonstrating the trend of gene prevalence percentages across different years, indicating how the presence of specific resistance genes has changed over time. **(D)** Regression of Average AMR Gene Count Over the Years: Scatter plot with a regression line showing the trend of average antimicrobial resistance (AMR) gene counts over the years, suggesting an increase or decrease in the accumulation of resistance genes in *C. difficile* isolates over time.

A comprehensive analysis of 19,711 *C. difficile* isolates revealed widespread and emerging antibiotic resistance patterns. Resistance genes were categorized into three frequency classes: high-frequency (>10% of isolates), medium-frequency (1-10% of isolates), and low-frequency (<1% of isolates) (**Figure 5A**). High-frequency genes, such as *cdeA* (99.46% of isolates), *cplR* (99.63%), and *nimB* (99.67%), were nearly ubiquitous, suggesting they confer significant survival advantages. Other notable high-frequency genes included *bla*_CDD-1_ (69.27%) and *bla*_CDD-2_ (30.32%). Genes associated with tetracycline and macrolide resistance, such as *tet(M)* (18.62%) and *ermB* (19.09%), indicated well-established resistance mechanisms.

Medium-frequency genes, like *ermQ* (0.2%) and *aph(2’’)-IIa* (0.18%), suggested emerging resistance, with *tet(O)* (0.39%) and *cfr(B)* (0.73%) indicating the potential for increased prevalence under selective pressure. While rare, low-frequency genes, such as *blaCTX-M-2*, *cfxA3*, *cfxA6*, and *blaZ* (each found in 0.005% of isolates), could pose future risks under certain conditions.

The antibiotic resistance (AMR) gene distribution between human and non-human hosts revealed shared and host-specific patterns (**Figure 5B**). Common AMR genes, such as *ccd-1*, *cdeA*, *cplR*, and *nimB*, were prevalent in both host groups, indicating their critical role in *C. difficile* survival across diverse environments. Host-specific genes included *aac(6’)-Ii*, *dfrE*, *efmA*, and *rpoB2*, found exclusively in non-human hosts at low prevalence (<0.1%). Conversely, *tet*(*T*), *vanR*, and *vanS* from the *vanA* cluster were detected only in human isolates, potentially linked to human-specific antibiotic exposure. Genes associated with reduced vancomycin susceptibility, particularly those from the *vanG* cluster, were found in both host groups at low frequencies (<0.1%), with a slight predominance in human hosts (24 isolates) compared to non-human hosts (five isolates), raising concerns about the potential for the spread of reduced vancomycin susceptibility. Additionally, *tet(M)* was more prevalent in non-human hosts (6.0%) than in human hosts (4.7%), suggesting higher selection pressure in non-human environments.

Gene presence patterns (GPPs) provided insights into the distribution of genes associated with resistance to critical antibiotics like metronidazole, vancomycin, and tigecycline (**Supplementary Figure 3**). Metronidazole resistance, mediated by *nim* genes, was observed in GPP 471 (associated with ST11) and GPP 462 (linked to ST48), with the presence of *nimB* indicating potential emerging resistance. Vancomycin resistance, mediated by *van* gene clusters, was identified in GPP 40 (associated with STs 15, 1, and 651) and GPP 173 (associated with ST3). Despite their low frequency, multiple *van* genes suggest robust resistance mechanisms in these patterns. The presence of van genes in isolates from 2007 to 2019 in the United States and the United Kingdom and their association with STs, such as ST1, ST3, ST21, ST63, and ST43, highlight the potential for the spread of reduced vancomycin susceptibility.

A broader analysis highlighted key antibiotic resistance patterns across the dataset. GPP 1, present in 50.2% of the isolates and associated with ST2, ST42, and ST8, was positive for *cdeA*, *cplR*, *nimB*, and *bla*_CDD-1_. GPP 2, found in 17.47% of the isolates and linked to STs1, ST55, and ST37, contained *tet(M)*, *ermB*, *bla*_CDD-2_, and *aph(3’)-IIIa*. GPP 3, observed in 3.48% of the isolates and associated with ST1, ST15, and ST54, included the genes *catA1*, *aad(6)*, *sat-4*, *tet(O)*, and *ant(6)-Ia*, demonstrating the prevalence and combinations of specific resistance genes within the *C. difficile* population and highlighting the complex landscape of antimicrobial resistance.

Temporal analysis (**Figure 5C**) revealed significant shifts in the prevalence of antibiotic-resistance genes over time. Genes like *cdeA* and *nimB* showed increased prevalence, indicating a growing resistance trend. However, regression analysis (**Figure 5D**) suggested that the overall increase in AMR genes per isolate was not statistically significant (R² = 0, p = 0.36), reflecting the dynamic nature of resistance evolution. Further analysis (**Supplementary Figure 4**) revealed that 97% of antibiotic resistance in *C. difficile* was chromosomal, with only 3% linked to plasmid-encoded genes. Chromosomal resistance was dominated by antibiotic inactivation (38.9%) and efflux pump mechanisms (38.1%), while on plasmids, efflux mechanisms (43.9%) and inactivation (24.6%) were significant, illustrating the bacteria’s capacity for rapid adaptation and resilience against antibiotics.

### 3.4 Prevalence and distribution of virulence factors in *C. difficile* isolates

**Figure 6:**
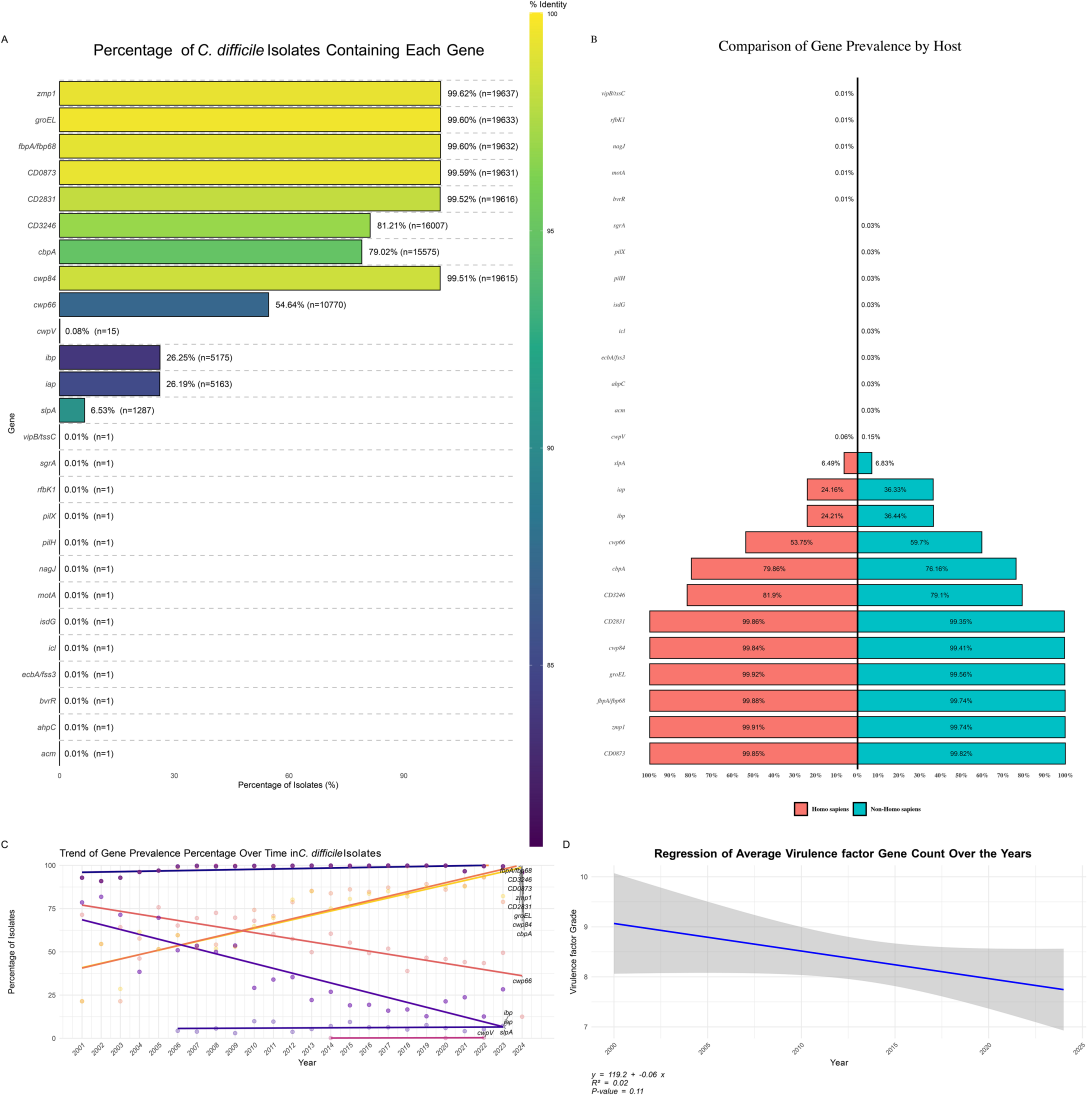
Distribution of Virulence Factor Genes in *C. difficile* Isolates. **(A)** Percentage of *C. difficile* Isolates Containing Each Virulence Factor: Bar plot illustrating the prevalence of specific virulence factor genes among *C. difficile* isolates. The color gradient represents the percentage of isolates containing each gene, highlighting the most and least common virulence factors. **(B)** Distribution of Virulence Factor Prevalence by Host: Bar plot showing the percentage of virulence factor gene prevalence grouped by different hosts, comparing the occurrence of these genes in human versus non-human hosts. **(C)** Trend of Virulence Factor Prevalence Percentage Over Time: Line plot demonstrating the trend of virulence factor gene prevalence percentages across different years, indicating how the presence of specific virulence genes has changed over time. **(D)** Regression of Average Virulence Factor Count Over the Years: Scatter plot with a regression line showing the trend of average virulence factor gene counts over the years, suggesting an increase or decrease in the accumulation of virulence genes in *C. difficile* isolates over time.

An analysis of *C. difficile* isolates revealed significant variability in the prevalence of specific virulence factor genes (**Figure 6A**). Highly prevalent genes, including *zmp1* (99.62%), *groEL* (99.60%), and *rpoB/rpoB2* (99.60%), were nearly universally present across the isolates. Other critical genes, such as CD0873 (99.57%), CD2831 (99.52%), and *cwp84* (99.51%), also showed high prevalence, underscoring their role in the pathogenicity and survival of *C. difficile*. Moderately prevalent genes, like *cwp66* (54.61%) and *slpA* (79.02%), exhibited a less consistent presence. Rare virulence factors, such as *cwpV*, *iapC*, and *motA*, were found in only 0.01% of the isolates, suggesting strain-specific or condition-dependent occurrence, highlighting the need for ongoing monitoring to understand their roles in pathogenicity.

The distribution of virulence genes between human and non-human hosts revealed similarities and differences (**Figure 6B**). Common virulence genes like CD0873, CD2831, *cwp84*, and *zmp1* were prevalent in 11% of isolates from both host types, indicating their essential role in *C. difficile* virulence across environments. The gene *cbpA* had a slightly higher prevalence in human isolates (9.1%) than in non-human isolates (8.5%), suggesting a broad distribution across host species. Some genes, like *cwp66*, were more common in non-human isolates (6.7%) than humans (6.1%). In contrast, others, such as *gap* and *bp*, were more prevalent in non-human hosts (4.1% vs 2.8% in humans), indicating possible environmental pressures or host-pathogen interactions. Certain genes, including *acm*, *ahpC*, *pilH*, *pilX*, and *sgrA*, were exclusive to non-human hosts but at very low prevalence (<0.1%). At the same time, *bvrR* and *motA* were found only in human isolates, suggesting host-specific virulence factors critical for *C. difficile* adaptation.

Trend analysis of virulence factor gene prevalence (**Figure 6C**) indicated a slight but consistent decline in the average number of virulence factor genes per *C. difficile* isolate from 2000 to 2024. The regression analysis (**Figure 6D**) suggested a negative trend (y = 119.2 - 0.06x), with a moderate correlation (R² = 0.32) but insignificant results (p = 0.11). This decline may reflect shifting evolutionary pressures on *C. difficile*, possibly due to changes in clinical practices, antibiotic use, and environmental factors influencing virulence trait selection.

The distribution of virulence factors genes presence patterns (GPPs) across *C. difficile* isolates (**Supplementary Figure 5**) revealed associations with specific sequence types (STs). The most prevalent pattern, GPP 1, included 37.43% of isolates, with eight genes commonly associated with ST2, ST42, and ST8, suggesting that these STs may dominate certain environments or host populations, contributing to widespread virulence. GPP 2, representing 23.96% of isolates, included nine positive genes linked to ST3, ST54, and ST35, highlighting their significant role in *C. difficile* virulence.

Other GPPs, such as GPP 3 (11.88%), GPP 4 (7.79%), and GPP 5 (4.29%), demonstrated the genetic diversity and complexity within *C. difficile* populations, suggesting multiple virulence strategies employed by different strains. Rare patterns like GPP 111 and GPP 112, representing only 0.01% of isolates, involved many positive genes, indicating specialized or emerging strains with unique virulence profiles, potentially marking new evolutionary paths or niche adaptations.

Further analysis identified various virulence genes, including *tcdE*, *tcdB*, *tcdR*, and *tcdA*, in isolates from countries collected between 2001 and 2023, associated with multiple STs. Among the isolates, 95.97% were toxigenic, possessing one or both toxin genes (*tcdA* and *tcdB*), while 4.03% were non-toxigenic. Specific STs, including ST15, ST747, ST3, ST83, and ST26, consistently tested positive for all genes except *tcdC*, suggesting these STs are likely highly toxigenic or pathogenic. These findings underscore the critical role of certain STs and gene presence patterns in shaping the virulence potential of *C. difficile* populations.

### 3.5 Prevalence and distribution of toxin genes in *C. difficile* isolates

**Figure 7:**
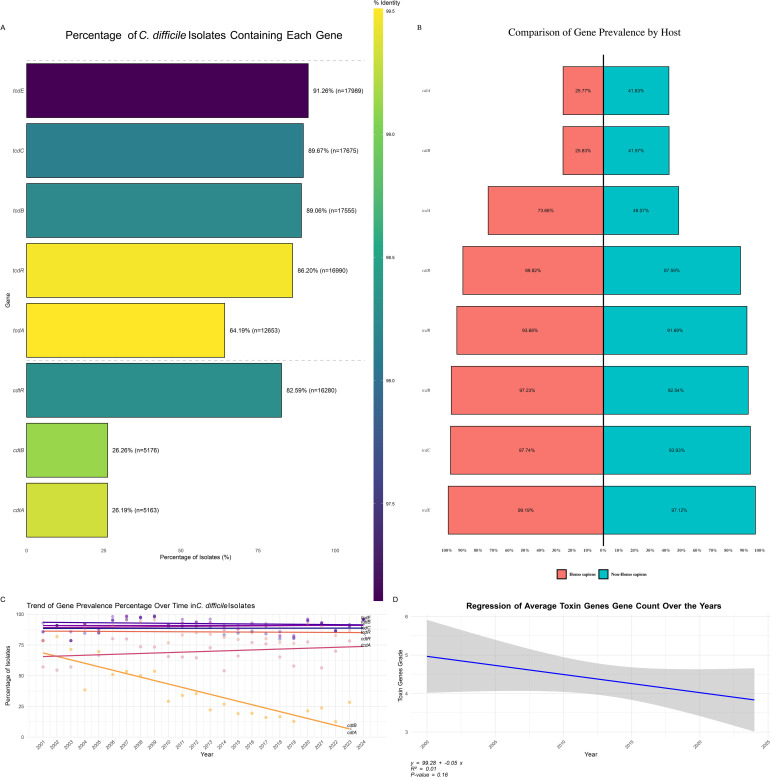
Distribution of Toxin Genes in *C. difficile* Isolates. **(A)** Percentage of *C. difficile* Isolates Containing Each Toxin Gene: Bar plot showing the prevalence of specific toxin genes among *C. difficile* isolates. The color gradient represents the percentage of isolates containing each toxin gene, highlighting the most and least common toxins. **(B)** Distribution of Toxin Gene Prevalence by Host: Bar plot depicting the percentage of toxin gene prevalence grouped by different hosts, comparing the occurrence of these genes in human versus non-human hosts. **(C)** Trend of Toxin Gene Prevalence Percentage Over Time: This is a line plot illustrating the trend of toxin gene prevalence percentages across different years, indicating how the presence of specific toxin genes has changed over time. **(D)** Regression of Average Toxin Gene Count Over the Years: Scatter plot with a regression line showing the trend of average toxin gene counts over the years, suggesting an increase or decrease in the accumulation of toxin genes in *C. difficile* isolates over time.

An analysis of toxin gene prevalence in *C. difficile* isolates revealed high occurrences of several key virulence factors (**Figure 7A**). The *tcdE* gene was the most prevalent, found in 91.26% of isolates (n = 17,989), followed by *tcdC* (89.67%, n = 17,675) and *tcdB* (89.06%, n = 17,555). These genes are widespread across the *C. difficile* population, underscoring their critical roles in pathogenicity. The regulatory gene *tcdR* was present in 86.20% of isolates (n = 16,990), while the major toxin gene *tcdA* was present in 64.19% of isolates (n = 12,653), indicating a somewhat lower but still significant prevalence.

In addition to the *tcd* genes, binary toxin genes were also assessed. The *cdtR* gene was found in 82.59% of isolates (n = 16,280), whereas *cdtB* and *cdtA* were less prevalent, found in 26.26% (n = 5,176) and 26.19% (n = 5,163) of isolates, respectively. These findings, depicted in **Figure 7B**, highlight the varying prevalence of different toxin genes within *C. difficile* populations, reflecting the bacterium’s diverse virulence strategies.

The host-specific distribution of toxin genes revealed distinct patterns between human and non-human hosts (**Figure 7C**). Binary toxin genes *cdtA* and *cdtB* were significantly more prevalent in non-human hosts (41.83% and 41.97%, respectively) than in human hosts (25.77% and 25.83%), suggesting these genes are more commonly retained in strains infecting non-human hosts, potentially due to differing environmental or selective pressures. Primary toxin genes, including *tcdA*, *tcdB*, *tcdC*, *tcdE*, and *tcdR*, were more prevalent in human isolates. Specifically, *tcdA* was present in 73.68% of human isolates and 48.07% of non-human isolates. *tcdB* was detected in 97.23% of human and 92.54% of non-human isolates, while *tcdC* and *tcdE* were more prevalent in human hosts (97.74% and 99.19%, respectively) compared to non-human hosts (93.93% and 97.12%). The regulatory gene *cdtR* was slightly more prevalent in human-associated strains (89.82%) compared to non-human isolates (87.59%).

Toxin gene presence patterns (GPPs) in *C. difficile* isolates, as shown in **Supplementary Figure 6**, revealed significant diversity in toxin gene profiles across different sequence types (STs). The most prevalent pattern, GPP1, observed in 42.78% of isolates, was associated with ST2, ST42, and ST8, and included six positive genes (*tcdR*, *tcdB*, *tcdE*, *tcdA*, *cdtR*, and *cdtB*), indicating that these STs harbor a comprehensive set of virulence factors contributing significantly to *C. difficile* pathogenicity. Other notable patterns, such as GPP 2 (16.38% of isolates) and GPP 3 (11.17%), involved different STs but also displayed a robust array of toxin genes. Conversely, rare patterns like GPP 10 (0.68%) and GPP 11 (0.44%), as well as GPP 70, GPP 69, and GPP 68, each representing only 0.01% of isolates, highlighted the genetic variability and potential niche specialization within the *C. difficile* population. This variation in gene expression across different GPPs underscores the complex evolutionary dynamics, with some patterns becoming dominant while others remain rare, potentially indicating emerging or declining strains.

Trends in toxin gene prevalence over time were also examined (**Figure 7C**). The *tcdE*, *tcdC*, *tcdB*, and *tcdR* genes exhibited relatively stable prevalence rates, consistently appearing in a high percentage of isolates throughout the study period. In contrast, *tcdA* showed a slight increase in prevalence, suggesting its rising presence in recent *C. difficile* strains. Interestingly, the binary toxin genes *cdtB* and *cdtA* demonstrated a marked decline in prevalence over time, indicating that these virulence factors may be losing prominence within the *C. difficile* population. This decline could reflect shifts in selective pressure, possibly due to changes in environmental conditions, host immune responses, or antibiotic use patterns favoring other virulence mechanisms.

Overall, the analysis of toxin gene trends provides critical insights into the evolving virulence landscape of *C. difficile*. A regression analysis of the average toxin gene counts (**Figure 7D**) indicated a slight decline in toxin gene prevalence from 2000 to 2024 (y = 99.28 - 0.05x). However, the R² value of 0.01 and a p-value of 0.16 suggest this trend is not statistically significant. This implies that while there was a downward trend, the change in toxin gene prevalence over time was minimal and may not significantly impact the overall virulence profile of *C. difficile* populations.

## 4 Discussion

Genomic analysis of *C. difficile* has provided significant insights into its pathogenicity, resistance mechanisms, and evolutionary dynamics (Knight et al., 2019). Our comprehensive examination covered various genomic aspects, including sequence length trends, antibiotic resistance genes, and virulence factors across different STs, each contributing uniquely to a bacterium’s behavior and adaptability.

One of the most striking findings of our analysis was the observed reduction in chromosomal sequence length over time. This trend, supported by a Pearson correlation coefficient of -0.37 (p < 0.001), suggests a significant evolutionary pressure toward genome re-sequencing in C. difficile. The tendency of more recent isolates to possess shorter genomes might reflect adaptive responses to selective pressures, such as antibiotic exposure and host immune defense (Knight et al., 2015). Genome streamlining typically involves the loss of essential genes and the reduction of intergenic regions. These improvements lead to more efficient genomes that can be replicated and maintained with fewer resources (Wolf and Koonin, 2013). This phenomenon is not exclusive to *C. difficile*; similar adaptations have been observed in other bacteria that evolved to thrive in specific niches or under continuous selective pressure (Giovannoni et al., 2014).

The analysis indicates that STs such as ST1 and ST11 are among the most frequently identified STs of *C. difficile*, consistently observed across several years, and highlights their enduring prevalence and adaptability (Dong et al., 2023). These observations are crucial for understanding the epidemiology of CDI and the potential for these strains to persist in healthcare settings and the community. Supporting this finding, an investigation by Abad et al., provides further evidence of the common isolation of these STs in the United States (Abad-Fau et al., 2023).

These findings suggest that ST1, ST11, and ST42 maintain their presence in various populations and exhibit a remarkable capacity to adapt to changing environmental conditions. The persistence of these STs across multiple years raises important questions regarding their evolutionary mechanisms and potential implications for public health. ST11, in particular, has been identified as a significant lineage associated with zoonotic transmission, with a notable prevalence in human and animal populations. This lineage has been linked to high antimicrobial resistance levels, which may contribute to its persistence and adaptability in various environments (Knight et al., 2019; Blau et al., 2023). Research indicates that ST11 is prevalent in clinical settings and agricultural contexts (Masarikova et al., 2020; Blasi et al., 2021). The emergence of AMR in ST11 strains, particularly against tetracycline and fluoroquinolone, poses a significant public health concern because these resistance traits can facilitate the survival of these strains in the presence of commonly used antibiotics (Blasi et al., 2021; Imwattana et al., 2021). The ongoing surveillance of ST11 and ST42 in American populations is imperative to address the challenges posed by these adaptable and resilient strains of *C. difficile*.

The contraction of chromosomal lengths in dominant *C. difficile* STs, particularly ST1, ST11, and ST42, highlights a potential evolutionary strategy to maintain essential functions while minimizing genetic load. This phenomenon is particularly evident when comparing isolates from around 2000, which often exceeded 4,300,000 base pairs, to those collected post-2015, which clustered around 4,100,000 base pairs. This reduction in chromosomal length suggests a trend toward genomic streamlining, where the loss of non-essential genes enhances the efficiency of bacteria in environments characterized by antibiotic exposure (Wolf and Koonin, 2013). This genomic specialization indicates the ability *of C. difficile* to evolve rapidly, optimizing its resistance mechanisms while maintaining core functionalities crucial for its persistence and virulence (Ramos-Silva et al., 2019). Understanding these evolutionary dynamics is vital for developing targeted strategies to combat antibiotic-resistant *C. difficile* strains and implementing public health interventions to control their spread (Muteeb et al., 2023). The box plot and heatmap illustrate the genomic characteristics of specific *C. difficile* STs, revealing that these STs exhibit more compact genomes than other lineages. This phenomenon, called genome streamlining, indicates evolutionary adaptation that enhances survival and competitiveness in ecological niches. The close clustering of these STs suggests high genetic similarity due to shared evolutionary backgrounds or environmental adaptations, reflecting evolutionary solid pressures (Turner et al., 2018).

In contrast, the size of plasmid sequences remained stable over time, with a correlation coefficient of -0.01 (p = 0.903). *C. difficile* plasmids primarily carry accessory genes that provide adaptive advantages under specific conditions, such as antibiotic resistance or additional virulence factors (Tijerina-Rodriguez et al., 2019). The stability of plasmid size suggests that these genetic elements have reached an optimal configuration that balances the benefits of carrying extra genes with the metabolic costs of maintaining and replicating larger plasmids (Dewan and Uecker, 2023).

In the ST analysis of *C. difficile*, specific STs, such as ST11, ST694, and ST167, they have exhibited distinct clustering patterns associated with various genetic characteristics influencing their pathogenicity and transmission dynamics. Clustering ST694 and ST167 alongside ST11 suggests that these lineages evolved in response to similar selective pressures, including antibiotic use, in agricultural and healthcare settings (Blau et al., 2023). Understanding these STs’ genetic relatedness and evolutionary history is crucial for developing effective strategies to combat *C. difficile* infections, particularly given the increasing incidence and severity of these infections across different populations (Hamo et al., 2021; Maikova et al., 2021). Horizontal gene transfer (HGT) and genetic recombination are fundamental mechanisms that enable *C. difficile* to adapt rapidly to changing environments (Knight et al., 2015). These processes allow bacteria to acquire advantageous genes from other organisms, such as those conferring antibiotic resistance and virulence. This enhances the adaptability of specific STs, enabling *C. difficile* to thrive in various environments, including healthcare facilities and agricultural settings (Oliveira et al., 2017). These STs appear to have undergone convergent evolution, possibly driven by selective pressures such as antibiotic exposure and immune challenges, resulting in their streamlined genetic architecture (Knight et al., 2019). The clustering patterns and temporal evolution of *C. difficile* STs reveal a dynamic adaptation process in response to antibiotic pressure (Weiss et al., 2021). The consistent presence of genes associated with reduced vancomycin susceptibility across key STs such as ST1, ST11, and ST42 underscores their significant role in the survival and pathogenicity of *C. difficile* in clinical environments (Abad-Fau et al., 2023). Our study identified various genes contributing to the bacterium’s reduced susceptibility to this critical antibiotic. Notably, the *vanG* operon emerged as a key player among the identified van gene operons, demonstrating a high degree of completeness and prevalence across the strains analyzed (Shen et al., 2020). This finding aligns with previous studies indicating that the *vanG* operon is often associated with the emergence of reduced vancomycin susceptibility in enterococci, suggesting a potential mechanism for transferring susceptibility traits within the *C. difficile* population (Knight et al., 2019). Although constituting a smaller portion of the genome, plasmids play a crucial role in rapidly acquiring and disseminating resistance traits in *C. difficile* (Vrancianu et al., 2020).

The present study also explored the frequency of virulence factor genes in *C. difficile*. Virulence factor genes, including essential toxins like *tcdA* and *tcdB*, are predominantly located on both chromosomes and plasmids and are consistently present on the chromosomes of various isolates (Awad et al., 2014; Di Bella et al., 2016). Over the past decade, there have been notable changes in the STs and toxin gene profiles of *C. difficile* (Knight et al., 2015). ST1 dominated until 2013, characterized by a complete set of toxin genes, but from 2014 to 2020, newer STs such as ST37, ST10, and ST203 emerged, with many losing the *cdtA* gene. Notably, ST203 lacks all toxin genes, indicating a shift from toxin-based pathogenicity to other survival strategies, reflecting the bacterium’s adaptability and underscoring the need for continuous monitoring (Knight et al., 2015). Plasmids are crucial extrachromosomal elements that carry genes contributing to antibiotic resistance and enhanced virulence (Hornung et al., 2019; Jasemi et al., 2020). The presence of variable accessory virulence genes across different isolates indicates that *C. difficile* can modify its virulence strategies depending on the environment or host conditions (Schuler et al., 2024). This adaptability is key to the survival and pathogenicity of the bacterium, allowing it to infect various hosts and thrive in diverse environments (Janoir et al., 2013). The current study emphasizes the importance of continuous genomic monitoring to track the emergence and spread of resistance and virulence factors, essential for effectively managing and controlling *C. difficile* infections (Vashisht et al., 2023). Incorporating genomic data into clinical and epidemiological strategies is crucial for developing targeted interventions and improving patient outcomes (Stark et al., 2019). Understanding the genetic basis of *C. difficile* pathogenicity and resistance will inform the development of new therapeutic approaches and guide public health policies to control its spread (Mengoli et al., 2022).

## 5 Conclusion

This study provides a comprehensive genomic analysis of *C. difficile*, revealing insights into its evolution, resistance mechanisms, and virulence factors by examining 19,711 whole-genome sequences. A key finding of this study is the trend toward genome scaling, characterized by reduced chromosomal sequence length, particularly in dominant STs, such as ST1, ST11, and ST42. This adaptation is driven by selective pressures, such as antibiotic exposure and host immune responses, and enhances survival in antibiotic-rich environments. Additionally, the study highlights the complex landscape of antibiotic resistance, particularly the persistence of genes such as *van* gene clusters, which contribute to reduced susceptibility to antibiotics like vancomycin, with the *vanG* operon consistently present across strains. Our analysis revealed a dynamic landscape of virulence factors in *C. difficile*, with critical toxins common among isolates. However, the emergence of STs with varying or absent toxin profiles indicates that the bacteria adapt their virulence strategies to different host environments under selective pressure.

## Data Availability

The original contributions presented in the study are included in the article/**Supplementary Material**. Further inquiries can be directed to the corresponding author.

## References

[B1] Abad-Fau A. Sevilla E. Martin-Burriel I. Moreno B. Bolea R. (2023). Update on commonly used molecular typing methods for clostridioides difficile. Microorganisms 11, 1752–1776. doi: 10.3390/microorganisms11071752 37512924 PMC10384772

[B2] Asempa T. E. Nicolau D. P. (2017). Clostridium difficile infection in the elderly: an update on management. Clin. Interv Aging 12, 1799–1809. doi: 10.2147/CIA.S149089 29123385 PMC5661493

[B3] Awad M. M. Johanesen P. A. Carter G. P. Rose E. Lyras D. (2014). Clostridium difficile virulence factors: Insights into an anaerobic spore-forming pathogen. Gut Microbes 5, 579–593. doi: 10.4161/19490976.2014.969632 25483328 PMC4615314

[B4] Blasi F. Lovito C. Albini E. Bano E. Dalmonte L. Drigo G.. (2021). Clostridioides difficile in calves in central Italy: prevalence, molecular typing, antimicrobial susceptibility and association with antibiotic administration. Anim. (Basel) 11, 515. doi: 10.3390/ani11020515 PMC7920295 33669325

[B5] Blau K. Berger F. K. Mellmann A. Gallert C. (2023). Clostridioides difficile from fecally contaminated environmental sources: resistance and genetic relatedness from a molecular epidemiological perspective. Microorganisms 11, 2497. doi: 10.3390/microorganisms11102497 37894155 PMC10608975

[B6] Boekhoud I. M. Hornung B. V. H. Sevilla E. Harmanus C. Bos-Sanders I. M. J. G. Terveer E. M.. (2020). Plasmid-mediated metronidazole resistance in Clostridioides difficile. Nat. Commun. 11, 598. doi: 10.1038/s41467-020-14382-1 32001686 PMC6992631

[B7] Botelho J. Schulenburg H. (2021). The role of integrative and conjugative elements in antibiotic resistance evolution. Trends Microbiol. 29, 8–18. doi: 10.1016/j.tim.2020.05.011 32536522

[B8] Buddle J. E. Fagan R. P. (2023). Pathogenicity and virulence of Clostridioides difficile. Virulence 14, 2150452. doi: 10.1080/21505594.2022.2150452 36419222 PMC9815241

[B9] Chen L. Zheng D. Liu B. Yang J. Jin Q. (2016). VFDB 2016: hierarchical and refined dataset for big data analysis–10 years on. Nucleic Acids Res. 44, D694–D697. doi: 10.1093/nar/gkv1239 26578559 PMC4702877

[B10] Dewan I. Uecker H. (2023). A mathematician’s guide to plasmids: an introduction to plasmid biology for modellers. Microbiol. (Reading) 169, 1752–1776. doi: 10.1099/mic.0.001362 PMC10433428 37505810

[B11] Di Bella S. Ascenzi P. Siarakas S. Petrosillo N. di Masi A. (2016). Clostridium difficile Toxins A and B: Insights into Pathogenic Properties and Extraintestinal Effects. Toxins (Basel) 8, 134–159. doi: 10.3390/toxins8050134 27153087 PMC4885049

[B12] Doll M. Marra A. R. Apisarnthanarak A. Al-Maani A. S. Abbas S. Rosenthal V. D. (2021). Prevention of Clostridioides difficile in hospitals: A position paper of the International Society for Infectious Diseases. Int. J. Infect. Dis. 102, 188–195. doi: 10.1016/j.ijid.2020.10.039 33122100

[B13] Dong Q. Lin H. Allen M. M. Garneau J. R. Sia J. K. Smith R. C.. (2023). Virulence and genomic diversity among clinical isolates of ST1 (BI/NAP1/027) Clostridioides difficile. Cell Rep. 42, 112861. doi: 10.1016/j.celrep.2023.112861 37523264 PMC10627504

[B14] Dureja C. Olaitan A. O. Hurdle J. G. (2022). Mechanisms and impact of antimicrobial resistance in Clostridioides difficile. Curr. Opin. Microbiol. 66, 63–72. doi: 10.1016/j.mib.2022.01.004 35077947 PMC9064893

[B15] Etifa P. (2021). The efficacy of probiotics in modulating Clostridium difficile spore germination, growth and toxin production in an *in vitro* human gut model. University of Hertfordshire.

[B16] Fitzpatrick F. Safdar N. van Prehn J. Tschudin-Sutter S. (2022). How can patients with Clostridioides difficile infection on concomitant antibiotic treatment be best managed? Lancet Infect. Dis. 22, e336–e340. doi: 10.1016/S1473-3099(22)00274-2 35617982

[B17] Giles J. Roberts A. (2022). Clostridioides difficile: Current overview and future perspectives. Adv. Protein Chem. Struct. Biol. 129, 215–245. doi: 10.1016/bs.apcsb.2021.11.003 35305720

[B18] Giovannoni S. J. Cameron Thrash J. Temperton B. (2014). Implications of streamlining theory for microbial ecology. ISME J. 8, 1553–1565. doi: 10.1038/ismej.2014.60 24739623 PMC4817614

[B19] Hamo Z. Azrad M. Fichtman B. Peretz A. (2021). The cytopathic effect of different toxin concentrations from different clostridioides difficile sequence types strains in vero cells. Front. Microbiol. 12. doi: 10.3389/fmicb.2021.763129 PMC8546354 34712220

[B20] Hornung B. V. H. Kuijper E. J. Smits W. K. (2019). An in silico survey of Clostridioides difficile extrachromosomal elements. Microb. Genom 5. doi: 10.1099/mgen.0.000296 PMC6807378 31526450

[B21] Imwattana K. Rodriguez C. Riley T. V. (2021). Knight DR. A species-wide genetic atlas of antimicrobial resistance in Clostridioides difficile. Microb. Genom 7, 7–20. doi: 10.1099/mgen.0.000696 PMC8743556 34793295

[B22] Janoir C. Deneve C. Bouttier S. Barbut F. Hoys S. Caleechum L.. (2013). Adaptive strategies and pathogenesis of Clostridium difficile from *in vivo* transcriptomics. Infect. Immun. 81, 3757–3769. doi: 10.1128/IAI.00515-13 23897605 PMC3811758

[B23] Jasemi S. Emaneini M. Fazeli M. S. Ahmadinejad Z. Nomanpour B. Sadeghpour Herav F.. (2020). Toxigenic and non-toxigenic patterns I, II and III and biofilm-forming ability in Bacteroides fragilis strains isolated from patients diagnosed with colorectal cancer. Gut Pathog. 12, 28. doi: 10.1186/s13099-020-00366-5 32518594 PMC7273666

[B24] Jia B. Raphenya A. R. Alcock B. Waglechner N. Guo P. Tsang K. K.. (2017). CARD 2017: expansion and model-centric curation of the comprehensive antibiotic resistance database. Nucleic Acids Res. 45, D566–D573. doi: 10.1093/nar/gkw1004 27789705 PMC5210516

[B25] Jolley K. A. Maiden M. C. (2010). BIGSdb: Scalable analysis of bacterial genome variation at the population level. BMC Bioinf. 11, 595. doi: 10.1186/1471-2105-11-595 PMC3004885 21143983

[B26] Knight D. R. Elliott B. Chang B. J. Perkins T. T. Riley T. V. (2015). Diversity and evolution in the genome of clostridium difficile. Clin. Microbiol. Rev. 28, 721–741. doi: 10.1128/CMR.00127-14 26085550 PMC4475645

[B27] Knight D. R. Kullin B. Androga G. O.. (2019). Evolutionary and Genomic Insights into Clostridioides difficile Sequence Type 11: a Diverse Zoonotic and Antimicrobial-Resistant Lineage of Global One Health Importance. mBio 10, 19–36. doi: 10.1128/mBio.00446-19 PMC6469969 30992351

[B28] Kouhsari E. Douraghi M. Fakhre Yaseri H. Talebi M. Ahmadi A. Sholeh M.. (2019). Molecular typing of Clostridioides difficile isolates from clinical and non-clinical samples in Iran. APMIS 127, 222–227. doi: 10.1111/apm.12937 30803047

[B29] Lewis B. B. Carter R. A. Ling L. Taur Y. Kamboj M. Dubberke E. R.. (2017). Pathogenicity locus, core genome, and accessory gene contributions to clostridium difficile virulence. mBio 8, 17–32. doi: 10.1128/mBio.00885-17 PMC5550754 28790208

[B30] Maikova A. Boudry P. Shiriaeva A. Vasileva A. Boutserin A. Medvedeva S.. (2021). Protospacer-Adjacent Motif Specificity during Clostridioides difficile Type I-B CRISPR-Cas Interference and Adaptation. mBio 12, e0213621. doi: 10.1128/mBio.02136-21 34425703 PMC8406132

[B31] Masarikova M. Simkova I. Plesko M. Eretova V. Krutova M. Cizek A. (2020). The colonisation of calves in czech large-scale dairy farms by clonally-related clostridioides difficile of the sequence type 11 represented by ribotypes 033 and 126. Microorganisms 8, 901. doi: 10.3390/microorganisms8060901 32549307 PMC7356540

[B32] Mengoli M. Barone M. Fabbrini M. D’Amico F. Brigidi P. Turroni S. (2022). Make It Less difficile: Understanding Genetic Evolution and Global Spread of Clostridioides difficile. Genes (Basel) 13, 13–31. doi: 10.3390/genes13122200 PMC9778335 36553467

[B33] Muteeb G. Rehman M. T. Shahwan M. Aatif M. (2023). Origin of antibiotics and antibiotic resistance, and their impacts on drug development: A narrative review. Pharm. (Basel) 16, 1615–1669. doi: 10.3390/ph16111615 PMC10675245 38004480

[B34] Nibbering B. Gerding D. N. Kuijper E. J. Zwittink R. D. Smits W. K. (2021). Host immune responses to clostridioides difficile: toxins and beyond. Front. Microbiol. 12. doi: 10.3389/fmicb.2021.804949 PMC8724541 34992590

[B35] Oliveira P. H. Touchon M. Cury J. Rocha E. P. C. (2017). The chromosomal organization of horizontal gene transfer in bacteria. Nat. Commun. 8, 841. doi: 10.1038/s41467-017-00808-w 29018197 PMC5635113

[B36] Page A. J. Cummins C. A. Hunt M. Wong V. K. Reuter S. Holden M. T. G.. (2015). Roary: rapid large-scale prokaryote pan genome analysis. Bioinformatics 31, 3691–3693. doi: 10.1093/bioinformatics/btv421 26198102 PMC4817141

[B37] Phanchana M. Harnvoravongchai P. Wongkuna S. Phetruen T. Phothichaisri W. Panturat S.. (2021). Frontiers in antibiotic alternatives for Clostridioides difficile infection. World J. Gastroenterol. 27, 7210–7232. doi: 10.3748/wjg.v27.i42.7210 34876784 PMC8611198

[B38] Ramos-Silva P. Serrano M. Henriques A. O. (2019). From Root to Tips: Sporulation Evolution and Specialization in Bacillus subtilis and the Intestinal Pathogen Clostridioides difficile. Mol. Biol. Evol. 36, 2714–2736. doi: 10.1093/molbev/msz175 31350897 PMC6878958

[B39] Schuler M. A. Riedel T. Overmann J. Daniel R. Poehlein A. (2024). Comparative genome analyses of clinical and non-clinical Clostridioides difficile strains. Front. Microbiol. 15. doi: 10.3389/fmicb.2024.1404491 PMC11238072 38993487

[B40] Seemann T. (2014). Prokka: rapid prokaryotic genome annotation. Bioinformatics 30, 2068–2069. doi: 10.1093/bioinformatics/btu153 24642063

[B41] Shen W. J. Deshpande A. Hevener K. E. Endres B. T. Garey K. W. Palmer K. L.. (2020). Constitutive expression of the cryptic vanGCd operon promotes vancomycin resistance in Clostridioides difficile clinical isolates. J. Antimicrob. Chemother. 75, 859–867. doi: 10.1093/jac/dkz513 31873741 PMC7069472

[B42] Sholeh M. Kouhsari E. Talebi M. Hallajzadeh M. Godarzi F. Amirmozafari N. (2021). Toxin gene profiles and antimicrobial resistance of Clostridioides difficile infection: a single tertiary care center study in Iran. Iran J. Microbiol. 13, 793–800. doi: 10.18502/ijm.v13i6.8081 35222857 PMC8816696

[B43] Sholeh M. Krutova M. Forouzesh M.. (2020). Antimicrobial resistance in Clostridioides (Clostridium) difficile derived from humans: a systematic review and meta-analysis. Antimicrob. Resist. Infect. Control 9, 158. doi: 10.1186/s13756-020-00815-5 32977835 PMC7517813

[B44] Stark Z. Dolman L. Manolio T. A. Ozenberger B. Hill S. L. Caulfied M. J.. (2019). Integrating genomics into healthcare: A global responsibility. Am. J. Hum. Genet. 104, 13–20. doi: 10.1016/j.ajhg.2018.11.014 30609404 PMC6323624

[B45] Szymczak J. E. Muller B. M. Shakamuri N. S. Hamilton K. W. Gerber J. S. Laguio-Vila M.. (2020). Prescriber perceptions of fluoroquinolones, extended-spectrum cephalosporins, and Clostridioides difficile infection. Infect. Control Hosp Epidemiol. 41, 914–920. doi: 10.1017/ice.2020.183 32468967

[B46] Tijerina-Rodriguez L. Villarreal-Trevino L. Morfin-Otero R. Camacho-Ortiz A. Garza-Gonzalez E. (2019). Virulence factors of clostridioides (Clostridium) difficile linked to recurrent infections. Can. J. Infect. Dis. Med. Microbiol. 2019, 7127850. doi: 10.1155/2019/7127850 31933709 PMC6942709

[B47] Turner C. B. Marshall C. W. Cooper V. S. (2018). Parallel genetic adaptation across environments differing in mode of growth or resource availability. Evol. Lett. 2, 355–367. doi: 10.1002/evl3.75 30283687 PMC6121802

[B48] Vashisht V. Vashisht A. Mondal A. K. Farmaha J. Alptekin A. Singh H.. (2023). Genomics for emerging pathogen identification and monitoring: prospects and obstacles. BioMedInformatics 3, 1145–1177. doi: 10.3390/biomedinformatics3040069

[B49] Vrancianu C. O. Popa L. I. Bleotu C. Chifiriuc M. C. (2020). Targeting plasmids to limit acquisition and transmission of antimicrobial resistance. Front. Microbiol. 11. doi: 10.3389/fmicb.2020.00761 PMC7219019 32435238

[B50] Weiss A. Lopez C. A. Beavers W. N. Rodriguez J. Skaar E. P. (2021). Clostridioides difficile strain-dependent and strain-independent adaptations to a microaerobic environment. Microb. Genom 7, 7–23. doi: 10.1099/mgen.0.000738 PMC8767335 34908523

[B51] Wolf Y. I. Koonin E. V. (2013). Genome reduction as the dominant mode of evolution. Bioessays 35, 829–837. doi: 10.1002/bies.201300037 23801028 PMC3840695

