# Supplementary material for: Global insights into the genome dynamics of Clostridioides difficile associated with antimicrobial resistance, virulence, and genomic adaptations among clonal lineages
Source: Front Cell Infect Microbiol. 2025 Jan 15;14:1493225. doi: 10.3389/fcimb.2024.1493225 (PMC11774869; doi:10.3389/fcimb.2024.1493225)
Supplement: Supplementary file 1 [file DataSheet1.docx]

**Supplementary File 2:**

**
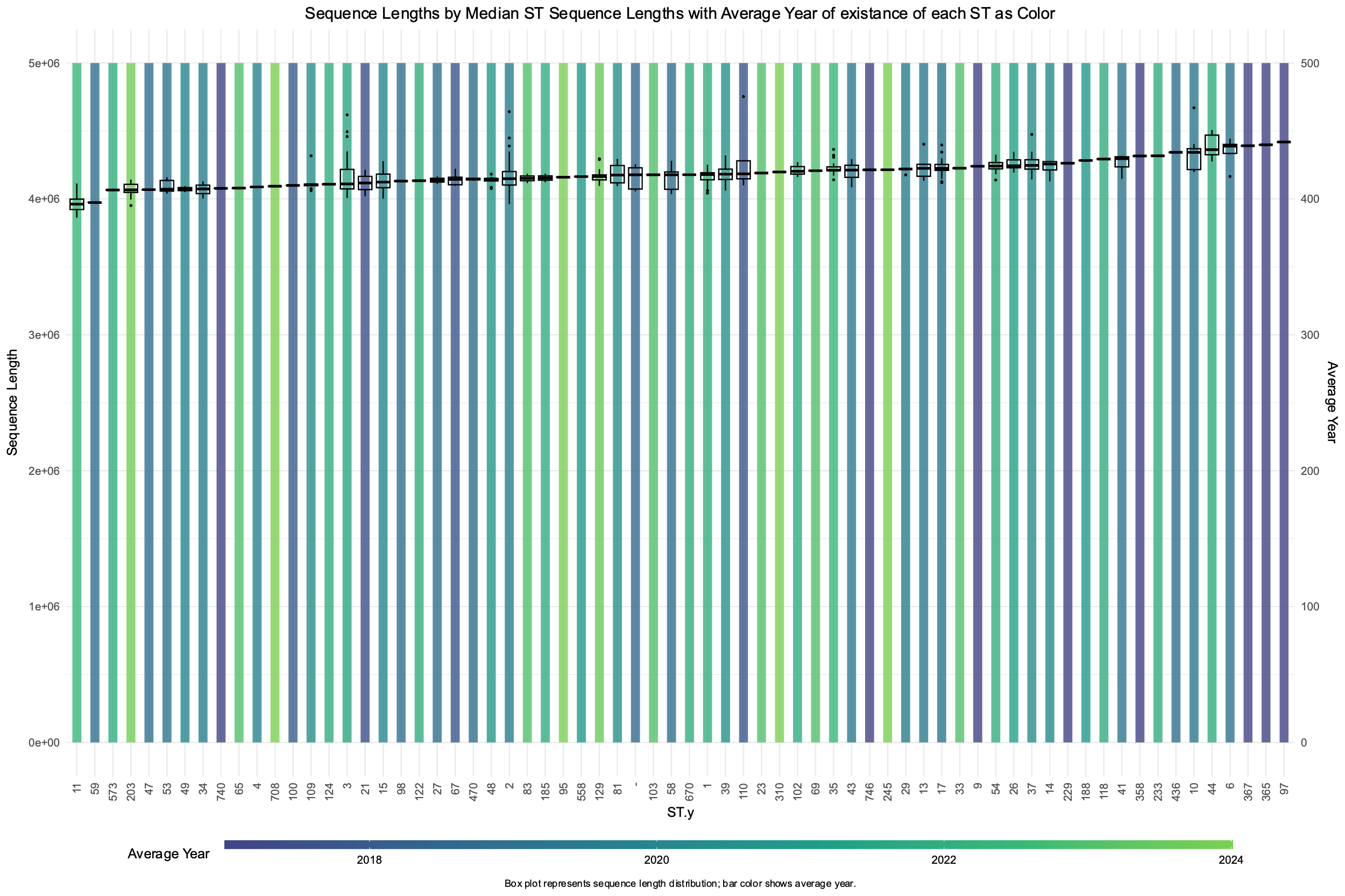
**

**Supplementary Figure 1:** Bar chart showing the distribution of average sequence lengths across different STs over time. The colour gradient indicates each ST's average year of existence, with a spectrum from earlier years (2000) to more recent years (2024). The box plots within each bar represent the variation in sequence lengths for each ST, emphasising how sequence lengths have evolved, particularly in response to environmental pressures and genetic streamlining.

**

**

**Supplementary Figure 2:** The roary plot of 239 *C. difficile* isolate genomes. This plot visualises the pan-genome of the isolates, displaying the presence and absence of genes across different strains. Each row represents a gene, and each column represents an isolate. The dark blue regions indicate the presence of specific genes in the isolates, while the light areas indicate their absence. The dendrogram on the left clusters the isolates based on the similarity of their gene content, highlighting the genetic diversity and shared genetic elements within the *C. difficile* population.

**Supplementary Figure 3: Antibiotic Resistance-related Genes Presence and Pattern of Them in** ***C. difficile***


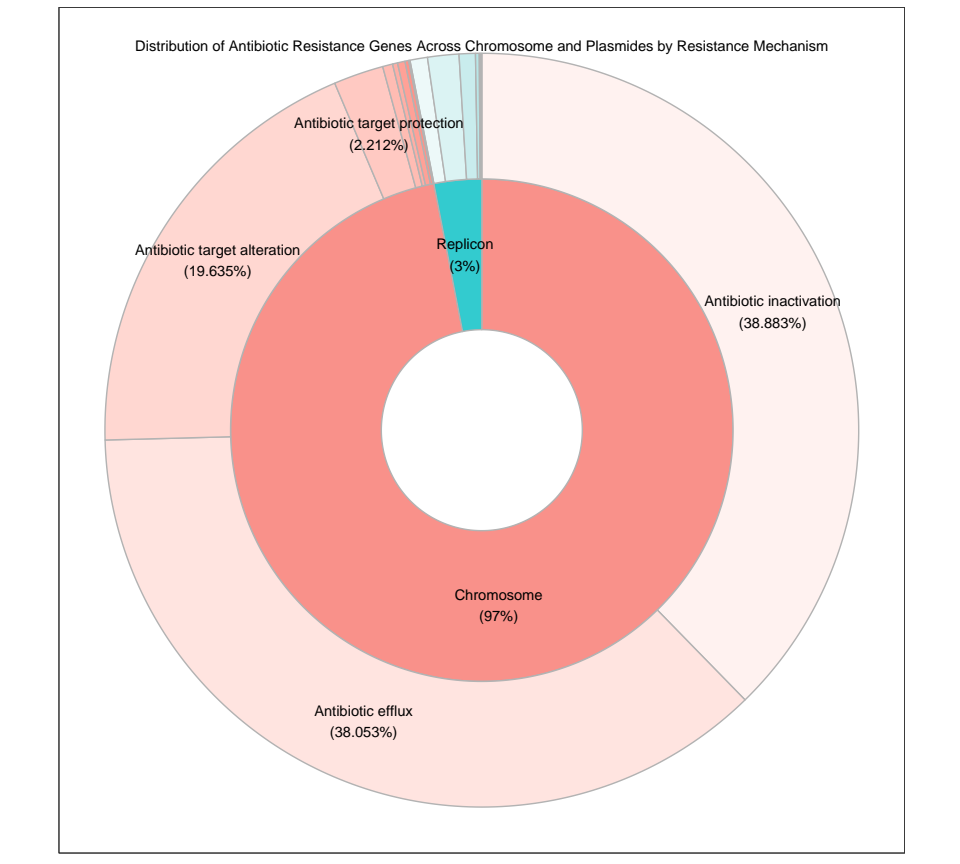


**Supplementary Figure 4:** Distribution of antibiotic resistance mechanisms in *C. difficile*

**Supplementary Figure 5: Virulence Factors Related Genes Presence and Pattern of Them in** *C. difficile*

**Supplementary Figure 6: Toxin Genes Presence and Pattern of Them in** *C. difficile*
